# New insights into prime Southern Ocean forage grounds for thriving Western Australian humpback whales

**DOI:** 10.1038/s41598-019-50497-2

**Published:** 2019-09-27

**Authors:** Sophie Bestley, Virginia Andrews-Goff, Esmee van Wijk, Stephen R. Rintoul, Michael C. Double, Jason How

**Affiliations:** 1 Institute for Marine and Antarctic Studies, University of Tasmania, Private Bag 129, Hobart, Tasmania 7001 Australia; 2 Australian Marine Mammal Centre, Australian Antarctic Division, 203 Channel Highway, Kingston, Tasmania 7050 Australia; 3 CSIRO Oceans and Atmosphere, GPO Box 1538, Hobart, TAS 7001 Australia; 4 Antarctic Climate and Ecosystems Co-operative Research Centre, Private Bag 80, Hobart, TAS 7001 Australia; 5 Centre for Southern Hemisphere Oceans Research, GPO Box 1538, Hobart, TAS 7001 Australia; 6 Department of Primary Industries and Regional Development, 140 William St, Perth, Western Australia 6000 Australia; 7 Western Australian Fisheries and Marine Research Laboratories, PO Box 20, North Beach, Western Australia 6920 Australia

**Keywords:** Animal migration, Marine biology

## Abstract

Humpback whale populations migrate extensively between winter breeding grounds and summer feeding grounds, however known links to remote Antarctic feeding grounds remain limited in many cases. New satellite tracks detail humpback whale migration pathways from Western Australia into the Southern Ocean. These highlight a focal feeding area during austral spring and early summer at the southern Kerguelen plateau, in a western boundary current where a sharp northward turn and retroflection of ocean fronts occurs along the eastern plateau edge. The topographic steering of oceanographic features here likely supports a predictable, productive and persistent forage ground. The spatial distribution of whaling catches and Discovery era mark-recaptures confirms the importance of this region to Western Australian humpback whales since at least historical times. Movement modelling discriminates sex-related behaviours, with females moving faster during both transit and resident periods, which may be a consequence of size or indicate differential energetic requirements. Relatively short and directed migratory pathways overall, together with high-quality, reliable forage resources may provide a partial explanation for the ongoing strong recovery demonstrated by this population. The combination of new oceanographic information and movement data provides enhanced understanding of important biological processes, which are relevant within the context of the current spatial management and conservation efforts in the Southern Ocean.

## Introduction

Humpback whale (*Megaptera novaeangilae*) migrations, between low-latitude winter breeding grounds and high-latitude summer feeding areas, represent one of the most extensive^1–3^ and consistent mammalian migrations known. For Southern Hemisphere populations, early knowledge of humpback whale (HBW) movements were derived from commercial catch distributions and basic *Discovery* tag mark-recaptures^4,5^. Further insight has developed from modern photo-identification and molecular methods^2,3,6–8^ and the current management of seven distinct Southern Hemisphere populations (A–G), under the International Whaling Commission (IWC), is increasingly informed by sophisticated genetic analyses (e.g.^9–13^).

Recovery patterns following intensive industrial whaling vary across populations, with differing levels of uncertainty, but the recent comprehensive assessment by the IWC Scientific Committee projected the Western Australian (WA) population to have the highest abundance, in the range of 18,415–24,918^14^. Western (breeding stock D) and Eastern Australian (breeding stock E1) populations have amongst the highest increase rates estimated, in the order of 9–12.7% (see^15^ and references therein) and 11.0%^16^, respectively. In contrast, the neighbouring Oceania population (comprising breeding stocks E2, E3, F1 and F2) remains listed as endangered by the International Union for Conservation of Nature^17^ and show no apparent recovery trend^18^ (but see also^19^). Factors controlling divergent population trajectories are unknown, and it is difficult to disentangle the historical harvest impact^20^ – including decimation from illegal hunting^21^ – from current environmental and/or anthropogenic factors. However, improved understanding of HBW feeding grounds may elucidate the role of factors such as migration length and energetic requirements in regulating recovery^22^, and provide an improved context for understanding likely impacts of future ecosystem changes^23–25^.

Links between specific breeding grounds and remote, relatively unsurveyed Antarctic feeding grounds remain poorly understood in many cases (e.g.^12,22^). Satellite telemetry is helping to clarify migratory routes, providing detailed information about key corridors, important habitats and potential for overlap with human activities^26–33^. Here, we present the first high-resolution migratory pathways to Southern Ocean summer forage grounds for the WA HBW population.

We show directed movements towards the southern Kerguelen plateau, a known area of high biological productivity^34–36^, including a focal usage of the western boundary current^37–39^ by both male and female HBWs. Using hidden Markov models, we discriminate sex-related movement behaviours, and examine residency behaviour in relation to current Southern Ocean management areas and a proposed Marine Protected Area. We consider this new information within the historical context of the spatial distribution of whaling catches, and what is known of other HBW movements in the region. We conclude that the southern Kerguelen plateau region is likely to provide a predictable, persistent and high-quality forage resource^36,37,40^, and usage of this area by WA HBWs may provide a partial explanation for the ongoing strong recovery of this population.

## Results

### General movement patterns

**Figure 1:**
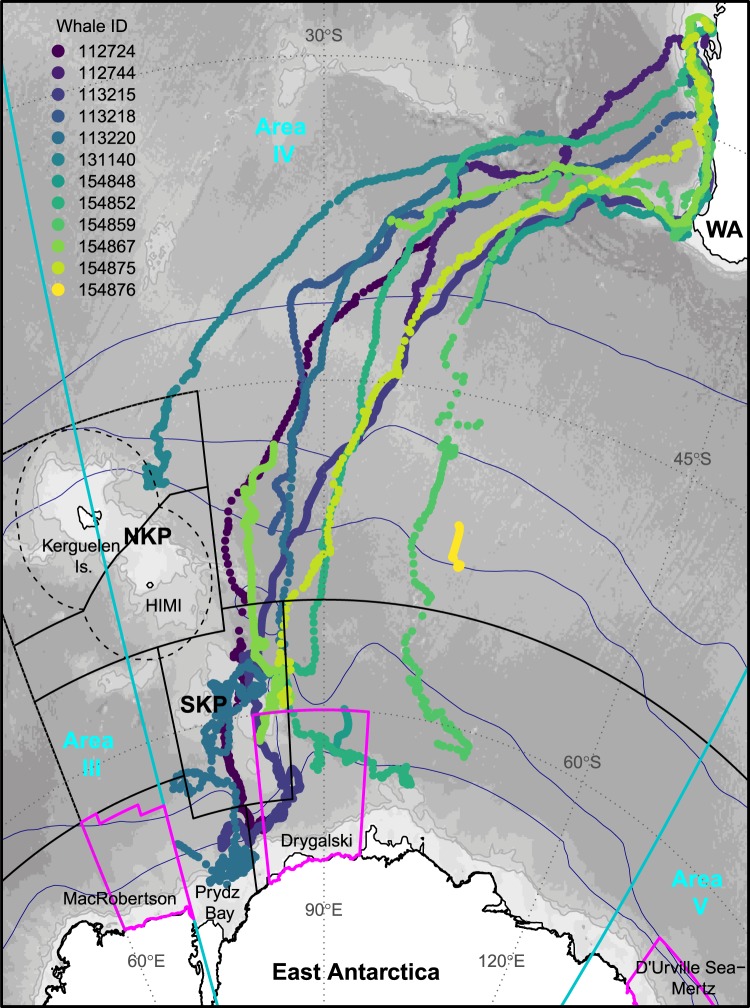
Southern Ocean migration pathways for humpback whales satellite-tracked off the western coast of Australia showing the full extent of southbound tracks. Individual whales (n = 12) are represented by colour. Relevant IWC Management Areas III, IV, and V, (demarcated at 70° and 130°E) are shown in turquoise. Three areas for an East Antarctic Representative System of Marine Protected Areas proposed by Australia, the European Union and its Member States are shown in magenta: MacRobertson, Drygalski and D’Urville Sea–Mertz. Black line vectors show the CCAMLR Statistical areas (https://gis.ccamlr.org/), and the Exclusive Economic Zones (dashed lines) around the Kerguelen Islands (France) and Heard Island and MacDonald Islands (HIMI, Australia). The climatological positions of the major ACC fronts^90^ are shown in navy. Background shading shows bathymetry^91^. Abbreviations: NKP – northern Kerguelen plateau; SKP – southern Kerguelen plateau; WA – Western Australia.

The migration pathways of the WA humpback whales tracked into the Southern Ocean (n = 12, Fig. 1) show whales left Australian coastal waters during the austral spring, generally between mid-September and early October (animation in Supplementary S2 shows the full Southern Ocean migrations). One third migrated south along the WA coastline, and travelled westward around 35°S from Australia’s southwestern corner into deeper waters over Naturaliste plateau, however an equal number departed from farther up the coast, including north of 30°S (Fig. 1). The remaining tags commenced reporting once offshore from WA.

HBWs migrated southwest towards the Kerguelen plateau in the Indian sector of the Southern Ocean, being tracked over total distances of 3551 to 11474 km (mean ± SD: 6680 ± 2620 km, Table 1). Based on the filtered state-space location estimates whales moved on average 69 ± 21 km day^−1^ (range: 28–107). One female headed to the northern Kerguelen plateau (ID131140); one continued in a more southward route towards the ice edge, staying east of 95°E (ID154859); only intermittent records appeared from ID154876 near 102°E, 52°S; and one tag failed during early transit (ID112744; at 96°E, 42°S). The other 8 out of 12 animals (67%) all travelled towards the southern Kerguelen plateau (SKP).

**Table 1:** Summary statistics for humpback whale spatial usage according to Southern Ocean management areas.

Individual whale ID	Sex^†^	Maximum displacement (km)	Total distance (km)	Total no. loc. (% Resident)	CCAMLR Statistical areas				Proposed Drygalski MPA	Northern
					58.5.1 Kerguelen	58.4.1 East Antarctic	58.4.2 Prydz Bay	58.4.3b Banzare		
112724	Male	4969	5298	451 (63%)		62 (84%)	7	286 (81%)	1	96 (0%)
112744	Unknown	2491	3551	42 (9%)						42 (10%)
113215	Male	5574	10300	2019 (79%)		480 (100%)	174 (66%)	975 (94%)	797 (95%)	390 (18%)
113218	Male	3743	4692	134 (0%)						134 (0%)
113220	Female	5401	11474	669 (66%)		5	170 (76%)	*395 (75%)		99 (0%)
131140	Female	4249	4284	205 (70%)	145 (99%)					60 (0%)
154848	Male	4430	^5752	79 (1%)		33 (0%)			30 (0%)	46 (0%)
154852	Unknown	4566	7854	305 (34%)		182 (58%)		25 (0%)	67 (36%)	98 (0%)
154859	Female	4020	8493	225 (47%)		107 (68%)				118 (25%)
154867	Unknown	4667	^8610	798 (85%)		28 (100%)		636 (97%)	120 (100%)	134 (21%)
154875	Female	4410	6075	134 (61%)		10 (40%)		18 (50%)		106 (10%)
**154876	Unknown	3598	^3777	53 (44%)						53 (47%)
**Overall**		**4343** **±** **834**	**6680** **±** **2620**	**5114** (**66%)**		**907** (**83%)**	**351** (**71%)**	**2335** (**89%)**	**1015** (**89%)**	**1376** (**12%)**

Distances (km) are reported from the tagging location (deployment details in Supplementary S1). Numbers indicate locations filtered at 6 h time steps, hence 4 locations per day. Numbers in parentheses indicate percent of locations in resident behavioural state (given where no. loc. ≥10). All WA tracking locations were within the range of 70.47 to 106.69°E, falling within the IWC management Area IV. ‘Northern’ column reports data outside (north of) the CCAMLR areas, noting the Hidden Markov Model (HMM) was fit to tracking data once south of 36°S to focus on Southern Ocean migrations. Resident state results are reported for the best supported HMM which included sex effects on state movement parameters (see Methods and electronic Supplementary Material S3). Abbreviations: MPA – Marine Protected Area.

^†^Determined by biopsy.

*Includes n = 3 (resident) locations from border of adjacent area 58.4.3a.

**Only reported Argos locations once south of 50°S.

^Total distance includes a net displacement calculated during a non-reporting period (see Table S1.1).

### Focus at the southern Kerguelen plateau

**Figure 2:**
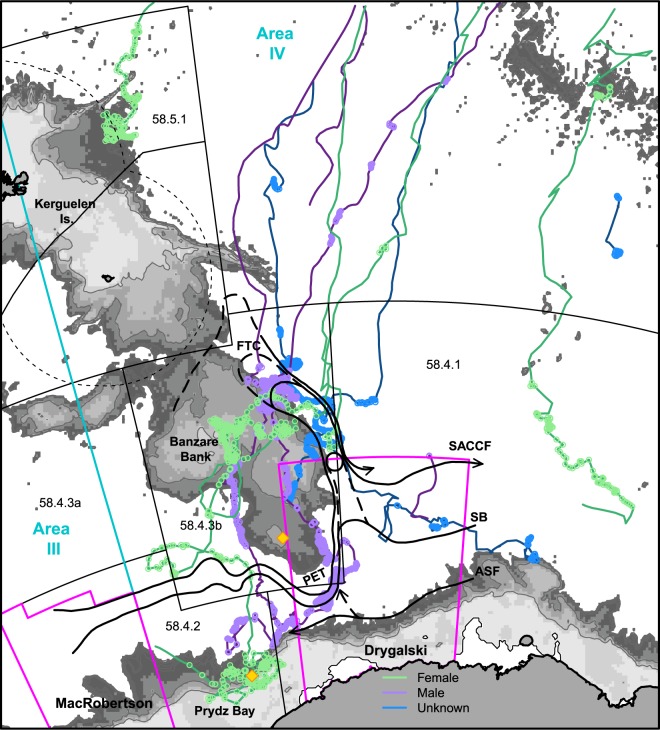
Migration pathways for satellite-tracked Western Australian humpback whales detailing the focus at the southern Kerguelen plateau. Tracks are coloured according to sex, with locations of inferred resident behavioural state shown as circles. Detailed oceanographic fronts^37^, as surveyed the summer prior during the 2016 KAXIS marine ecosystem survey, are shown as bold black lines. Acoustic listening stations (yellow diamonds) were located at Prydz Bay (2013) and the southern Kerguelen plateau (2014–2017) as part of the Southern Ocean hydrophone network^92^. Background shading shows bathymetry^91^ truncated at 3000 m. The numbering indicates CCAMLR Statistical areas; all other detail represented as in Fig. 1. Abbreviations: PET – Princess Elizabeth Trough; ASF – Antarctic Slope Front, SB – Southern Boundary, SACCF – Southern ACC Front, FTC – Fawn Trough Current.

First arrival to the SKP area occurred at the end of October and start of November, approximately 6 weeks after leaving Australian coastal waters (42 ± 7 days, range: 31–49 days). Tracks of six individual whales show a notable focus along the eastern flank of the SKP, between the Southern Boundary and the southern Antarctic Circumpolar Current (ACC) front (Fig. 2 and animation, Supplementary S2). Here, the topographic steering turns these fronts sharply to the north before they retroflect southward again and continue meandering eastward^35,37,39^. Figure 2 shows the fronts as mapped during a marine ecosystem voyage undertaken the previous summer (January–February 2016)^37^. The real-time dynamic height contours associated with these features are shown in Supplementary S2 (animation presents daily satellite oceanographic and sea ice data from September 2016 to March 2017). This important western boundary current^39^ is known to advect subpolar waters and sea ice northward, extending sea-ice extent and duration in this region^35^.

Whales continued to occupy this frontal area and Banzare Bank during November and December, well after the sea-ice has retreated southward (animation, Supplementary S2). One subadult individual (ID154852) did follow the retreating ice, demonstrating movements in and around the ice edge during this time. Tracks from whales which continued into January 2017 (IDs 112724, 113215, 113220 and 154867) show movements southward from Banzare Bank, into the Princess Elizabeth Trough and the north of Prydz Bay. Two whales (ID113215 and ID113220) show movement in and around the sea ice in this region during February-March, until these tags failed as the sea-ice began advancing again in mid-late March.

### Sex-related movement behaviours

The best supported hidden Markov model for Southern Ocean humpback whale movements included sex effects on the behavioural state movement parameters (step length and turn angle concentration), but not on the state transition probabilities (Supplementary S3, Table S3.1). Once south of Australia (i.e., beyond 36°S) whales spent 66% (CI: 65–68%) of their time in ‘resident’ behaviour, although with substantial variability among individuals (Table 1). Overall transition probabilities between states were low (i.e., Pr(Resident|Transit) = 0.035 [0.025, 0.049] and Pr(Transit|Resident) = 0.017 [0.011, 0.024]; results presented as estimate [lower, upper 95%CI]).

The results indicate female movements were substantially faster, particularly during transit but also in the resident state (Supplementary S3, Fig. S3.1, Table S3.2). The estimated gamma distributions for step length during transit had means (*µ*) of 5.15 km h^−1^ for females (CI: 4.78–5.51), in comparison to 3.16 km h^−1^ (CI: 2.94–3.38) for males and 4.00 km h^−1^ (CI: 3.69–4.30) for unsexed animals. The movement estimates for the resident state were much lower overall (females: 2.15 km h^−1^ [1.92–2.38]; males: 0.89 km h^−1^ [0.85–0.93]; unsexed: 0.85 km h^−1^ [0.79–0.90]). Across sexes, more directed movements were evident from mean turn angles (λ) close to zero during transit, whereas values close to $\pi$ indicate higher turning rates during resident phases. Similarly, across sexes the turn angle distributions show higher concentration parameters (*Κ*) during transit, albeit this appeared rather weaker for males (females: 0.62 [0.56–0.68]; males: 0.38 [0.31–0.44]; unsexed: 0.60, CI: 0.53–0.66). Across sexes the concentration parameter estimates were generally close to zero during residency (Supplementary S3, Table S3.2).

### Movement behaviour in relation to spatial management areas

All tracking locations fall within the IWC management Area IV (tracking location range: 70.47–106.69°E), with the last location estimate for female ID113220 being at the Area III boundary (70.47°E, 64.87°S) just prior to tag failure (Fig. 2). Whale movements traversed CCAMLR Statistical areas 58.4.1 (7), 58.4.2 (2), 58.4.3b (5) and 58.5.1 (1) (Fig. 2), with substantial resident periods within these areas identified from between 1 and 7 whales (as shown in brackets). Summarised dive data available for ID131140 in the vicinity of the northern Kerguelen plateau (area 58.5.1) shows active usage of the upper water column (Supplementary S4). Over 5,000 dives were recorded over approximately 14 reporting days, with mean maximum dive depths of 83 ± 38 m. The largest number of locations (approximately 584 d total) and highest residency rates (mean: 89%) were in Statistical area 58.4.3b at Banzare Bank (Table 1). The four whales with the longest tag life (records that continued past mid-December 2016) all logged locations here. In relation to the Drygalski area of the proposed EARSMPA: four tracked whales reported substantially from this area (>1 week), two of which exhibited very high residency rates (i.e., ≥95%), with one male resident here for just over four months.

### Broader spatiotemporal context

**Figure 3:**
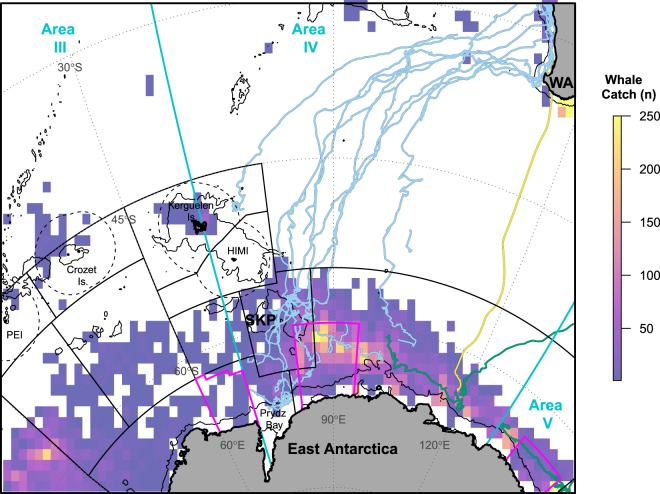
Southern Ocean migration pathways for satellite-tracked WA humpback whales overlaid with historical whaling catches. Whale tracks (n = 12) from Fig. 1 are shown in light blue. Two additional female East Australian whales (IDs 88729 tagged in 2008 and 96386 tagged in 2010^32^) which moved westward are shown in green. A single Southern Ocean migration available from a female WA whale (ID96382 tagged in 2009^41^) tracked during an earlier deployment is shown in yellow. Historical catch data shows the total number of individual HBWs reported to the International Whaling Commission (IWC) aggregated per 1 × 1° grid cell (see Methods). IWC Management Areas III, IV, and V, demarcated at 70° and 130°E, are shown in turquoise. CCAMLR Statistical areas (black boxes), national EEZs in the subantarctic (black dashed lines), and three areas proposed for the East Antarctic MPA (magenta, bold boxes) are also indicated. Bathymetry contour^91^ shows the 2000 m isobath. Abbreviations: WA – Western Australia, SKP – southern Kerguelen plateau, PEI – Prince Edward Islands, HIMI – Heard Island and MacDonald Islands.

The spatial distribution of whaling catches (Fig. 3) supports the importance of the SKP region for humpback whales since at least historical times. Total catches above 200 individuals per 1 × 1° IWC reporting grid cell occur in the vicinity of the SKP, with high catches occurring here over a wide latitudinal band and generally contracting south towards the Antarctic continent farther east. Tracking data from two female East Australian humpback whales^32^ confirm some movement occurs into Area IV from the east; one of these females spent time near 120°E before moving as far west as 108°E, where the track terminated. One WA female from an earlier deployment^41^ migrated quite directly south, also towards 120°E, further suggesting some overlapping of foraging ranges for Eastern and Western humpback whales. Reported whaling catches west of the SKP are comparatively low until the Weddell gyre. Sparse tracking records from eastern South African HBW (stock C) have recently documented movement towards the Crozet and Prince Edward Islands, and westward of 30°E (data in^28,30^; not shown).

## Discussion

The detailed migration pathways presented here enrich our knowledge of the long-distance migrations undertaken by WA humpback whales and their usage of important Southern Ocean seasonal feeding grounds. This new information is relevant within the context of the current spatial management and conservation efforts to protect marine areas in the East Antarctic/Indian Ocean sector. While our findings are consistent with existing general knowledge of HBW movement patterns, the high-resolution biotelemetry reveals specific usage of the western boundary current at the southern Kerguelen plateau. The high productivity around the SKP, controlled by the interaction of topography and oceanography, is likely to provide a predictable, persistent and high-quality forage resource for whales^36,37,40^.

Simplified movement patterns of HBWs derived from early Discovery mark recoveries show a clear connectivity between the low-latitude WA breeding grounds and high-latitude feeding grounds in the Indian sector of the Southern Ocean^4,5,42^. The spatial distribution of whaling catches (Fig. 3) further supports the importance of this region to HBWs since at least historical times (*i.e*., as documented from the 1930s). In fact blue whale (*Balaenoptera musculus*) catches show a very similar spatial distribution in this sector^43^, and regional survey sightings of humpback, blue, sperm (*Physeter macrocephalus*) and minke (*B. cutorostra*) whales^40,44^ indicate the southern Kerguelen plateau is important to other baleen and toothed whales. Ship-based survey sightings highlight cetacean densities around the southern and eastern flanks of the SKP^40^. Both catch and survey data show generally high cetacean densities extend farther northward (i.e., over a broader latitudinal extent) directly east of the southern Kerguelen plateau^43–45^.

These cetacean distributions reflect the overall patterns of regional biological productivity and oceanographic processes^43–45^. The phytoplankton bloom southeast of the SKP has been shown to be persistent throughout the summer^34,35^ with a number of interplaying mechanisms hypothesized to support this sustained productivity, including: topographic upwelling, ice edge blooms, the advection of biomass and/or nutrients from the south, and potentially gyre retention (see discussions by Sokolov and Rintoul^34^ and Rintoul and colleagues^35^). The regional surface ocean circulation and frontal structure, including sharp northward turn and retroflection of the fronts in a western boundary current along the eastern plateau margin (Fig. 2 and^37^), was described during a recent marine ecosystem voyage (Kerguelen Axis, KAXIS) the summer prior to our main tracking dataset. The topographic steering of currents dictates both the southern extent of the ACC and the northern limit of subpolar waters, which likely influences the distribution and productivity of marine ecosystem at multiple trophic levels^37^.

Examining KAXIS dissolved iron measurements within the broader context of *in situ* and satellite chlorophyll-*a* concentrations, Schallenberg and co-authors^36^ concluded the regional ocean circulation plays an important role in supplying the iron necessary to sustain these persistent blooms. The northward horizontal advection of subsurface iron, likely originating from Antarctic shelf sediments in the south, and subsequent upwelling – along the eastern margin of the SKP and in association with frontal processes – combine to create a biological hotspot. This new research clarifying biophysical linkages provides a deepened understanding of the importance of this feeding ground to migrating HBWs, where the conditions support predictable and persistent high biological productivity. We speculate that this feeding scenario may be more favourable than using more dispersed and/or ephemeral resources, such as oceanic or marginal-ice zone foraging. The bathymetric control of currents and regional productivity might also be more resilient to change.

The location of the SKP feeding grounds additionally appears to result in relatively direct travel pathways for WA HBWs. For example, WA HBW net displacements approached a maximum of around 5500 km from the tagging location (Table 1), showing quite directed movements to their feeding area, along defined migratory corridors, with consistent movements between individuals. This may translate directly as a feeding migration that provides a ‘good bang for the buck’, or return for the effort invested, especially when viewed in comparison to some migration patterns recorded for other HBW populations. For example, migratory pathways recently documented for Oceania HBWs^33^ show highly dispersed trajectories, spread across around 4500 km of Antarctic feeding grounds. Notably, of these Oceania HBWs the tagged mother-calf pairs showed the shortest migrations, moving more directly south to the Ross Sea (~4500 km). Adults without dependant calves predominantly moved into the Bellingshausen and Amundsen Seas, showing that minimum straight-line distances over 6000 km are not exceptional^2^. Other extreme long-distance HBW movements (>8000 km) can occur between feeding areas along the Antarctic Peninsula and breeding areas off Central America^1^.

It is worth noting that neither study (WA nor Oceania HBWs) tagged whales on the breeding grounds, but in both cases the total migratory pathway includes substantial movement from breeding sites to the tagging locations. On the basis of photo identification and genetic matches HBWs passing the Kermadec Islands did not assign to a single Oceania breeding ground origin, but instead came from a range of breeding grounds spanning ~3500 km of ocean^33^. Reported straight-line distances from assigned breeding grounds ranged from ~900 km (Tonga, breeding stock E3) to ~2000 km (Cook Islands, stock F1), and included assigned origins at New Caledonia (~1700 km, stock E2), Niue (~1300 km) and American Samoa (~1800 km). French Polynesia is also a breeding site for Oceania HBWs^46^ therefore a straight line distance of 3000–3500 km is also a possibility. HBWs have a breeding ground preference for shallow coastal waters and bays^46,47^ and recent work has expanded the recognized calving grounds for the WA HBW population (breeding stock D) along the extensive coastline of north-western Australia, from Camden Sound in the Kimberley (15°S) to at least North West Cape (22°43’S)^48^. Of those WA HBWs that we tracked into the Southern Ocean, only one was tagged on its northbound trip. This individual showed a northernmost terminus (turning point) at 114.93°E, 20.38°S (Table S1.2), approximately 600 km beyond where most southbound WA HBWs were tagged (these deployment sites span latitudes 21.75°S–25.48°S, see Table S1.1). The tracking data available for other northbound WA HBWs tagged under this same study (Table S1.2) reveal outermost turning points ranging from 100–500 km south (26.28°S) of our tag deployment locations to one movement as far northwest as ~1900 km (13.70°S). In general, however, recorded turning points were 872 ± 268 km (range: 173–1396 km) past our tag deployment sites. Comparative data from previous deployments^49^ were 1220 ± 443 km (range: 228–1771 km). Overall, these investigations suggest that WA breeding ground movements are likely to be of a similar, or somewhat smaller, scale than the comparable movements of Oceania HBWs.

The energetic costs of these different migration distances remain to be evaluated in comparison to the energetic benefits obtained from favourable feeding grounds, and/or favourable breeding grounds (including developmental conditions for offspring, plus favourable conditions for energy conservation for lactating females, i.e. in resting areas)^47^. It seems likely that these three important elements are together potentially facilitating the strong recovery of the WA population^15,22^: the availability of suitable breeding, calving and resting areas along the WA coast^47^, a relatively direct migration (compared to other HBW populations), and temporally persistent high-quality prey in a productive boundary-current ecosystem.

The consistency of the SKP feeding ground as a predictable, persistent and high-quality forage resource for HBWs, matches with the WA (stock D) population being identified as mainly “classical feeders” from isotopic analyses^50^. Baleen plates from a small number of stranded WA HBWs (n = 7) showed isotopic profiles consistent with continual provisioning, or dietary provenance, from a low trophic-level Antarctic prey source. In comparison, isotopic profiles of 13 East Australian (stock E1) animals showed this population was more likely to feed outside the traditional Antarctic zone; for example, with supplementary temperate feeding as has been regularly reported^51–53^. Recent satellite tracking analyses also indicate supplemental feeding during residencies in south-eastern Australian and New Zealand waters, supported by direct visual observations of feeding off Eden at the time of tagging^32^.

Within the isotopic signatures that assigned WA HBWs as “classical feeders”, Eisenman and colleagues^50^ observed temporal increases in δ^15^N decoupled from δ^13^C. Such changes could reflect spatial variability in the δ^15^N signatures of Antarctic prey, and/or variability of isotopic values at the base of the food web (e.g. pelagic vs sea-ice diatoms^54^). Alternatively, such changes might indicate shifts to a higher trophic level of Antarctic feeding, either by the prey or by the WA HBWs (albeit in insufficient quantities to exit their Antarctic δ^13^C isotope range estimated using average values for Antarctic krill, *Euphausia superba*). The spatial distribution of the WA HBWs tracked at the SKP shows a focus quite far north in the western boundary current (57–60°S). Additionally, a single adult female HBW was resident for more than 1 month at the northern KP (~48°S) with dive data showing very active vertical movement during this period (Supplementary S4). Given this, the intriguing question arises of whether these whales are foraging north of the putative Antarctic krill biome; and if so, what are WA HBWs likely to be feeding upon?

With respect to the spatial extent of the krill biome, an empirical relationship derived for krill growth rate in relation to chlorophyll and temperature has recently been applied across the Southern Ocean^55,56^. This approach suggests that by December the SKP area (80–110°E, 50–60°S) in fact represents high potential growth rates for 40 mm krill (>3 mm month^−1^)^55^. This high-growth habitat contracts farther southward towards the Antarctic continent by January^55^. The SKP western boundary current may therefore represent one of the locations where winter ice extent (overwintering krill larvae) coincides with high adult potential growth rates during summer, enabling the krill lifecycle to be completed successfully (^55^, their Fig. 6c). However the krill net tow data^57^ necessary to confirm this hypothesis are only typically available from scientific research voyages conducted during mid-summer (January–February, as in KAXIS) and closer to the Antarctic continent^58,59^. Similarly, the IWC’s comprehensive circumpolar whale sightings surveys were all conducted south of 60°S^60,61^. Since whaling operations favour low ice conditions, spatial distributions drawn from historical whale catches may also bias towards the Antarctic continent in summer. Spatial distributions derived from catches are also confounded by many other conditions (*e.g*., distance to the nearest port, ice dynamics, weather, recent catch experience). Therefore, to properly test this hypothesis of a northward extension in krill – and hence whale – habitat in this region it would be necessary to conduct research voyages with net tows during late spring and early summer (November to early December).

With respect to the question regarding likely alternative prey, for bulk-feeding baleen whales swarming or schooling organisms are the most lucrative food resource^62^. Globally, the known prey of HBWs include a wide assortment of schooling fish including herring (*Clupea* spp.) and sand lance (*Ammodytes* spp.); temperate shrimp, amphipods, and coastal krill (*Nyctiphanes australis*); as well as euphausiid species such as the lipid-rich *E. superba*^51–53^. HBWs are known for the variety and complexity of their feeding behaviours, with capture techniques that include bubble nets, tail slaps, lunges and bottom feeding^63^. Alternate candidate Antarctic prey items that have higher δ^15^N signatures might include aggregations of other euphausiids such as *Thysanoessa macrura* or *E. triacantha*^64^. The distribution *T. macrura* overlaps that of *E. superba* but may extend to relatively warmer water temperatures; they are also a known prey for whales, and can be very abundant, locally replacing *E. superba* as the most abundant euphausiid^65^ and references therein. Other candidate prey might include amphipods (e.g. *Themisto gaudichaudii*^66^) or possibly small mesopelagic fishes such as *Krefftichthys anderssoni*^67^. Diving data during the HBW residency (ID131140) at the northern Kerguelen plateau are indicative of active foraging in the upper 150 m of the water column (Supplementary S4). The Kerguelen Plateau, extending from a theoretically krill-dominated food-web in the south^68^ to a more copepod-fish dominated food-web in the north^69,70^ potentially offers migrating HBWs diverse and rich food opportunities.

Southern Hemisphere humpback whale populations are characteristically high in genetic diversity^9,10^, thought to be maintained by substantial reproductive sub-structuring across breeding and feeding populations^14^. The extent to which mixing between neighbouring populations occurs is poorly known. However, regional-scale contact between, and interchange of, individuals is assumed to take place primarily on shared feeding areas in the Southern Ocean^10,11^. Discovery mark-recaptures imply that HBWs from WA (stock D) are likely to mix with individuals from eastern Australia (stock E1), particularly via east-to-west movements on the feeding grounds^5^ (and^42^, their Fig. 3.6). Few Discovery marks were ever deployed in Area III (n = 119) but two Antarctic deployments near 10°E and 60°E, recovered at Madagascar, do indicate significant longitudinal movements^4,42^. Satellite tracking from eastern South Africa remains similarly scant, showing truncated migratory journeys in Area III towards the Crozet and Prince Edward Islands, as well as westward of 30°E^28,30^. As increasing high latitude genetic sampling (mitochondrial DNA) is becoming available, mixed stock analyses are being used to determine the likely population origins of whales across Southern Ocean feeding grounds^11,71,72^. Such an analysis considering stocks C, D and E would be required to describe the population structure on the SKP feeding ground and evaluate the possibility of this being a shared high-quality feeding ground.

Future population assessments considering feeding ground density dependence have also been recommended^14^. It is unknown to what extent prey availability, as a consequence of habitat quality in the Southern Ocean, may provide a constraint on HBW population numbers. Our analysis of movements on the forage grounds, showing significantly higher movement rates by females during both transit and residency, further raises interesting questions regarding the energetic requirements and benefits between the sexes. It is important to note that we did not tag mother-calf pairs which tend to move more slowly^33,41^. Hence, the difference in movement rates might be simply explained by size differences with the larger body size of females enabling faster travel relative to males. Alternatively, with high pregnancy rates recently reported in southern hemisphere HBWs^73^ it is possible these females were pregnant, driving high energetic requirements and hence greater foraging effort. Larger sample sizes (tracking and biopsy) would be useful to resolve these points.

Early work proposed the Southern Boundary of the ACC to be of circumpolar ecological importance to the productivity of Southern Ocean ecosystems and the baleen whales which depend upon them^43^. Since then, the interaction of the ACC flow with topography is increasingly understood to influence physical and biological processes at local, regional and large scales^74^. The new detailed migratory pathways for western Australian humpback whales highlight the southern Kerguelen plateau as a key feeding area. Recent marine survey efforts here have improved our understanding of how the high regional productivity, structured by the interaction of ocean currents and topography, is likely to support a predictable, persistent and rich supply of prey resources for whales. From a management perspective, HBWs and other baleen whale populations are the concern of the IWC^14^. Alongside, the Convention on the Conservation of Antarctic Marine Living Resources (CAMLR Convention) applies to Antarctic marine living resources in the Convention area, which is divided into nine marine protected area planning domains^75^. Here, CCAMLR has agreed to establish a representative system of marine protected areas, which seek to address a suite of conservation objectives. Specifically, the foraging migrations of many species of penguins, seals and flying seabirds are considered in the MPA planning and proposal processes, and these data, for example, contributed directly to the Drygalski area of the East Antarctic (and other) MPA proposal(s)^76^. While this proposed MPA does encompass an important HBW forage area in the southeast, the key forage area associated with the western boundary current clearly extends farther to the north. As we turn towards the future it seems most likely that these two management interests of IWC and CCAMLR will converge in the arena of risk assessments for krill fishing in the Southern Ocean^77^. In particular, as CCAMLR works to manage harvesting of krill and other species with a precautionary approach this shall be achieved in a way that accounts for consumption needs across predators, including those managed by the IWC, at spatially meaningful scales.

## Methods

### Tracking data

The tracking data presented here were collected as part of research efforts to reduce fishing gear entanglements in Australian waters (see also^78^) as humpback whales migrate north and south within coastal fishery areas. Tagging was approved and conducted under the Australian Department of the Environment Cetacean Permit 2014-0005 and the Western Australian Department of Parks and Wildlife (DPAW) permits SF009946 and SF010439. All tagging methods were carried out in accordance with the relevant guidelines and regulations. Full details of tag types and deployment methods are given in Supplementary S1, Table S1.1. From these coastal deployments, 12 tagged whales provide longer tracking records that document southward migrations into the Southern Ocean (n = 18,597 Argos-relayed locations in total; tracking longevity typically 49–192 days). These data represent mostly adults and available biopsies indicate both sexes (Female: 4, Male: 4, Unknown: 4 including one subadult). This project did not tag mother-calf pairs. A small amount of information on vertical movements is available for two whales that were equipped with WC SPLASH10-260C satellite and dive logger tags (Supplementary S1 and S4).

### Data processing – location filtering

Due to the irregular timing and errors associated with Argos location data, these were filtered using a state-space model (SSM^79^), to obtain a regular time sequence of location estimates with reduced uncertainty. The SSM used is a variant of the widely applied model of ^80^, as described in^81^ and^82^ theirAppendix S2. This fast implementation uses the R package TMB^83^. Associated R and C++ code for processing the Argos data are publicly available as an R package *ssmTMB* version 0.0.1.9100 (https://github.com/ianjonsen/ssmTMB). The SSM was fit with a 6 h time step providing four location estimates per day (n = 6,348 total). Step lengths (speeds) and turning angles were calculated from these filtered locations and provided to the Hidden Markov Models (HMMs) described below.

### Data analysis – hidden markov model application

For inferring (latent) animal behaviours from movement tracks we applied discrete-time hidden Markov models (HMMs^84–86^). Specifically focussing on the Southern Ocean movements (i.e., once south of Australia beyond 36°S; n = 5114 locations) we investigated 2-state model formulations to discriminate behaviours nominally termed ‘transit’ and ‘resident’. Typically during transit whales are expected to undertake more directed movements, at generally higher speeds of travel and with lower turning rates; the converse occurs during more resident periods, which may occur in areas with higher foraging returns^32,87^. However movement may differ between sexes^29^. To examine for sex-related movement patterns, we investigated four different 2-state HMM configurations: (1) a basic 2-state model, with (2) sex-dependent transition probabilities between states, (3) sex-dependent movement parameters and (4) both sex-dependent transition probabilities and movement parameters. In all models, we used a gamma distribution for step length and a wrapped Cauchy distribution for turning angles, with the model parameters estimated across individuals using a maximum likelihood approach implemented with the R package *momentuHMM* version 1.4.2^86^. A multiple imputation approach was employed that enables pooled inferences across the HMM analyses to reflect location uncertainty^86^. Model parameters are calculated as pooled estimates (across *n* = 100 realizations of the location estimates) and 95% confidence intervals (Supplementary S3).

### Historical whaling catches

Historical catch data for Southern Ocean humpback whales were obtained from the IWC^88^. We compiled all reported southern hemisphere HBW catches between 0–150°E per 1 × 1° grid cell (n = 71,120). HBW catch records south of 36°S within the region 50–150°E (n = 18,481) span the pre- and post-war periods 1929–1939 and 1946–1968. West of 50°E, available catch records (n = 8,187) span a longer period (1883–1904, 1918–19, 1928–1973).

### Southern Ocean management

We report whale movements within the spatial context of: the relevant IWC Management Areas (Area III/IV boundary is classified at the meridian of longitude 70°E and Area IV/V boundary at 130°E); the Commision for the Conservation of Antarctic Living Marine Resources (CCAMLR) Statistical Areas (https://gis.ccamlr.org/home/ccamlrgis); and with reference to the relevant area(s) within the East Antarctic Representative System of Marine Protected Areas (EARSMPA) jointly proposed to CCAMLR by Australia, the European Union and it’s Member States^89^.

## Supplementary information

Electronic Supplementary Material S1-S4

Supplementary S2. Tracking animation.

## Data Availability

The satellite tracking data used in this study is held in the National Marine Mammal Database managed by the Australian Marine Mammal Centre (https://data.marinemammals.gov.au/nmmdb).
