# Supplementary material for: New insights into prime Southern Ocean forage grounds for thriving Western Australian humpback whales
Source: Sci Rep. 2019 Sep 27;9:13988. doi: 10.1038/s41598-019-50497-2 (PMC6764985; doi:10.1038/s41598-019-50497-2)
Supplement: Supplementary file 1 — Electronic Supplementary Material S1-S4 [file 41598_2019_50497_MOESM1_ESM.pdf]

Supplementary Material for:

New insights into prime Southern Ocean forage grounds for thriving Western Australian humpback whales

Sophie Bestley[1*], Virginia Andrews-Goff[2], Esmee van Wijk[3,4], Stephen R. Rintoul[3,4,5], Michael C. Double[2] and Jason How[6,7]

[1] Institute for Marine and Antarctic Studies, Private Bag 129, University of Tasmania, Hobart Tasmania 7001 Australia

[2] Australian Marine Mammal Centre, Australian Antarctic Division, 203 Channel Highway, Kingston, Tasmania 7050 Australia

[3] CSIRO Oceans and Atmosphere, GPO Box 1538, Hobart, TAS 7001, Australia

[4] Antarctic Climate and Ecosystems Co-operative Research Centre, Private Bag 80, Hobart, TAS 7001, Australia

[5] Centre for Southern Hemisphere Oceans Research, GPO Box 1538, Hobart, TAS 7001, Australia

[6] Department of Primary Industries and Regional Development, 140 William St, Perth, Western Australia 6000 Australia

[7] Western Australian Fisheries and Marine Research Laboratories, PO Box 20 North Beach Western Australia 6920 Australia

* Corresponding author email: Sophie.Bestley@utas.edu.au

SUPPLEMENTARY INFORMATION

Supplementary material for this article includes:

Supplementary S1. Tag deployment details.

Supplementary S2. Tracking animation.

Supplementary S3. Hidden Markov model results

Supplementary S4. Dive logger data available for two migrating WA humpback whales.

SUPPLEMENTARY S1 – TAG DEPLOYMENT DETAILS

Western Australian humpback whales (n = 62) were satellite-tagged on their northward migration (June-July) of 2015 and 2016 and on their southward migration (September) of 2014 and 2016 as part of research related to fishery entanglements in coastal Australian waters (see also How et al. 2015). Of these, 12 tags recorded migratory pathways of whales into the Southern Ocean (Table S1.1).

Humpback whales were tagged with a number of different biotelemetry devices, all blubber penetrating satellite tags. Short tags (22 cm, n = 6) housed SPOT 177N satellite tags from Wildlife Computers [WC], Redmond, Washington, United States of America. Long tags (30 cm) housed either a Kiwisat 202 by Sirtrack Havelock North, New Zealand (n = 3); a WC SPOT 193J (n = 1); or a WC SPLASH10-260C satellite and dive logger tag (n = 2).

The whales were tagged from a 5.45 m fiberglass rigid-hull inflatable vessel equipped with a modified tagging bowsprit, at distances of about 2-6 m. A biopsy sample was also taken when possible to determine the sex of the individual (as per Double et al. 2012). All 12 whales were considered in good condition at the time of tagging by visual assessment of body dimensions (i.e. avoiding any individuals with visual evidence of impaired health). This project did not tag mother-calf pairs.

The tags relay estimates of geographic location via satellite using the Argos System (Collecte Localisation Satellites, Toulouse, France) with each location assigned an estimated error and one of seven associated location classes (CLS Argos, 2016). Location processing (n = 18,597 total) and subsequent analyses are described in the Methods. For the two tags with a non-reporting period (Table S1.1), filtering and modelling procedures treated data as separate segments.

All tagging was carried out under the Australian Department of the Environment Cetacean permit number 2014-0005, with ethical approval from the Western Australian Department of Parks and Wildlife (DPAW permits SF009946 & SF010439). The full project details are available in the Fisheries Research and Development Corporation Final Report 2014/004 (How et al. *in prep*).

References

CLS Argos. 2016. Argos user's manual, Collecte Localisation Satellites, Toulouse France. http://www.argos-system.org/manual/.

Double, M.C., Jenner, K.C.S., Jenner, M-N., Ball, I., Childerhouse, S., Laverick, S., Gales, N. 2012. Satellite tracking of northbound humpback whales (*Megaptera novaeangliae*) off Western Australia. Australian Marine Mammal Centre Final Report May 2012, Australian Antarctic Division, Hobart Australia. 23pp.

How, J., Coughran, D., Smith, J., Double, M., Harrison, J., McMath, J., Hebiton, B., A Denham, A. 2015. Effectiveness of mitigation measures to reduce interactions between commercial fishing gear and whales. FRDC Project No 2013/03. Fisheries Research Report No. 267. Department of Fisheries, Western Australia. 120pp.

How, J., Coughran, D., Smith, J., Double, M., Harrison, J., McMath, J., Hebiton, B., A Denham, A. *in prep*. Mitigation measures to reduce entanglements of migrating whales with commercial fishing gear. FRDC Project No 2014-004 Fisheries Research Report. Department of Fisheries, Western Australia.

Irvine, L. G., Thums, M., Hanson, C. E., McMahon, C. R. & Hindell, M. A. 2018. Evidence for a widely expanded humpback whale calving range along the Western Australian coast. *Mar Mamm Sci* **34**, 294-310, doi:10.1111/mms.12456.

Table S1.1. Tag deployment details for humpback whales (n = 12) satellite-tagged in Western Australian waters which provided records of Southern Ocean feeding migrations. Abbreviations: for sexed animals determined from biopsies: F – female, M – male; else U – unknown; tag position: L – left, R – right.

Tag	Date	Time	Lon [°E]	Lat [°S]	Sex	Maturity	Tagging location	Tag Type	Tag position	Migration	Longevity (days)	No. Argos Locations*
112744	5/09/2014	08:35	113.56	24.82	U	Adult	Carnarvon	WC SPOT-177N	L	South	49	1001
154859	30/06/2016	10:32	115.18	34.36	F	Adult	Augusta	WC SPOT-177N	L	North†	149	837
154852	6/09/2016	11:40	113.52	24.94	U	Subadult	Carnarvon	WC SPOT-177N	L	South	101	2505
154867	6/09/2016	13:22	113.50	24.92	U	Adult	Carnarvon	WC SPOT-177N	R	South	129^	1831
154876	12/09/2016	12:23	114.15	21.75	U	Adult	North West Cape	WC SPOT-177N	R	South	13	45**
113215	13/09/2016	14:50	114.17	21.90	M	Adult	North West Cape	Kiwisat 202	R	South	192	3067
154848	15/09/2016	09:15	113.52	24.99	M	Adult	Carnarvon	WC SPOT-177N	R	South	81^^	682
154875	15/09/2016	10:15	113.49	25.05	F	Adult	Carnarvon	WC SPLASH10-260C (TDR)	L	South	57	684
113220	15/09/2016	10:59	113.47	25.07	F	Adult	Carnarvon	Kiwisat 202	R	South	170	3348
131140	15/09/2016	11:25	113.47	25.07	F	Adult	Carnarvon	WC SPLASH10-260C (TDR)	R	South	63	1302
113218	21/09/2016	12:43	113.02	25.48	M	Adult	Carnarvon	Kiwisat 202	R	South	52	288
112724	21/09/2016	14:15	113.02	25.48	M	Adult	Carnarvon	WC SPOT-193J	R	South	113	3007

*Excludes all duplicate time and/or near duplicate Argos location/time records

**Only reported Argos locations once south of 50°S (from 23/11)

^ No locations transmitted during the 28 day period 31/10 to 28/11

^^ No locations transmitted during the 17 day period 24/10 to 1/11

† Individual ID154859 was tagged on the northbound trip. Distance calculations reported in Table 1 of main manuscript are relative to the location 112.29°E, 25.27°S (i.e. once animal was again southbound) as at 21/08/2016

Table S1.2. Northern terminus (turning point) information recorded by satellite-tagged humpback whales along the north-western coastline of Western Australian. Turning points are calculated at the northernmost extent of the northbound migrations. Data shown where available for whales tagged during this study in 2015-2016 (n = 11; How et al. *in prep*) as well as from previous deployments during 2008 and 2011 (n = 7; Double et al. 2012). According to Irvine et al. (2018), the calving grounds for the Breeding Stock D population extend south from Camden Sound in the Kimberley (15°S) to at least North West Cape (22°43'S), 1,000 km southwest of the previously recognized calving area between Camden Sound and Broome (15–18°S). Abbreviations: for sexed animals determined from biopsies: F – female, M – male; else U – unknown

Tag	Date	Lon [°E]	Lat [°S]	Sex	Tagging location
This study					
131169	02/08/2015	118.56	19.78	F	Augusta
131170	13/07/2015	112.51	26.28	U	Augusta
131165	29/07/2015	121.88	17.82	M	Augusta
131167	21/08/2015	119.80	19.43	U	Augusta
154857	27/07/2016	121.48	18.95	F	Augusta
154855	07/08/2016	115.35	19.69	M	Augusta
154860	01/08/2016	119.04	19.57	F	Augusta
154872	07/08/2016	126.41	13.70	F	Augusta
154853	31/07/2016	119.75	19.24	M	Augusta
154874	16/08/2016	122.36	16.92	F	Augusta
154859	31/07/2016	114.93	20.38	F	Augusta
Previous	***deployments***				
86583	08/08/2008	122.88	15.22	M	Broome
86584	07/08/2008	123.64	15.55	M	Broome
86586	06/08/2008	123.60	15.27	U	Broome
53398	07/08/2011	124.75	14.44	M	Ningaloo Reef
98107	24/07/2011	116.60	20.29	F	Ningaloo Reef
98127	24/07/2011	115.32	20.02	M	Ningaloo Reef
98140	10/08/2011	122.39	15.16	M	Ningaloo Reef

SUPPLEMENTARY S2 – TRACKING ANIMATION

Animation showing humpback whales satellite-tracked from Western Australia in relation to daily mapped satellite sea surface currents and sea-ice concentration within the Indian sector of the Southern Ocean during 1 September 2016 to 31 March 2017.

Whale tracks. Tracking data shown for individual whales represent 6 hourly location estimates obtained from a state-space model used to filter Argos locations (see Methods).

Sea surface height and geostrophic currents. Background displays the magnitude of geostrophic current velocities (m s^{-1}) derived from daily sea gridded maps of absolute dynamic topography (MADT, m). The MADT products were produced by Ssalto/Duacs and distributed by Aviso at 0.25° x 0.25° resolution, with support from the Centre national d'études spatiales (CNES) (http://www.aviso.altimetry.fr/duacs/). This system processes data from all altimeter missions to provide consistent products.

Dynamic height contours from daily maps of absolute sea surface height (SSH) provide information on the broad regional circulation and the behaviour of the eastward flowing Antarctic Circumpolar Current (ACC) as it encounters the Kerguelen Plateau (Bestley et al. 2018; Kim and Orsi, 2014; Sokolov and Rintoul, 2009a, b; van Wijk et al. 2010). Hydrographic, satellite-based and high-resolution modelling studies have all demonstrated that the major ACC fronts in fact consist of multiple branches (see Sokolov and Rintoul, 2009a and references therein). Here, we provide a few diagnostic markers. Following Bestley et al. (2018) in all images we mark SSH contours at −1.15 m (red, southernmost) and −1.10 m (orange) which are co-located with their hydrographic interpretations of the Southern Boundary (SB) and Southern ACC Front (SACCF) during the KAXIS survey, 2016. These streamlines are steered south of the southern Kerguelen plateau, through the Princess Elizabeth Trough, and subsequently show dynamic extensions northward along the plateau's eastern flank. The −1.00m streamline (yellow) corresponds well with the mean position of a northern branch of the SACCF (Bestley et al. 2018; Sokolov and Rintoul, 2009a, b). We additionally show the 0 m (black) streamline as a marker for the core of the ACC's Subantarctic Front (Kim and Orsi, 2014) as well as −0.80 (magenta) for the vicinity of the southern branch of the Polar Front (PF-S, see Bestley et al. 2018 and references therein).

Sea Ice. Daily ice concentrations (%) are shown in greyscale with white (grey) indicating high (low) ice. Daily passive microwave estimates of sea-ice concentration (%) were obtained from the National Snow and Ice Data Center SMMR-SSM/I polar product available for the Southern Hemisphere gridded at 25 km resolution (Cavalieri et al., 1996, updated yearly; Maslanik and Stroeve, 1999, updated daily).

The bathymetric 1000 and 2000 m isobaths are highlighted (grey contours, Weatherall et al. 2015) as are the acoustic listening stations (yellow diamonds) located at Prydz Bay (2013) and the southern Kerguelen plateau (2014-2017) as part of the Southern Ocean hydrophone network (Miller and Miller, 2018). CCAMLR Statistical areas (gray-blue boxes) and the relevant areas of the proposed East Antarctic Representative System of Marine Protected Areas (cyan boxes) are also indicated.

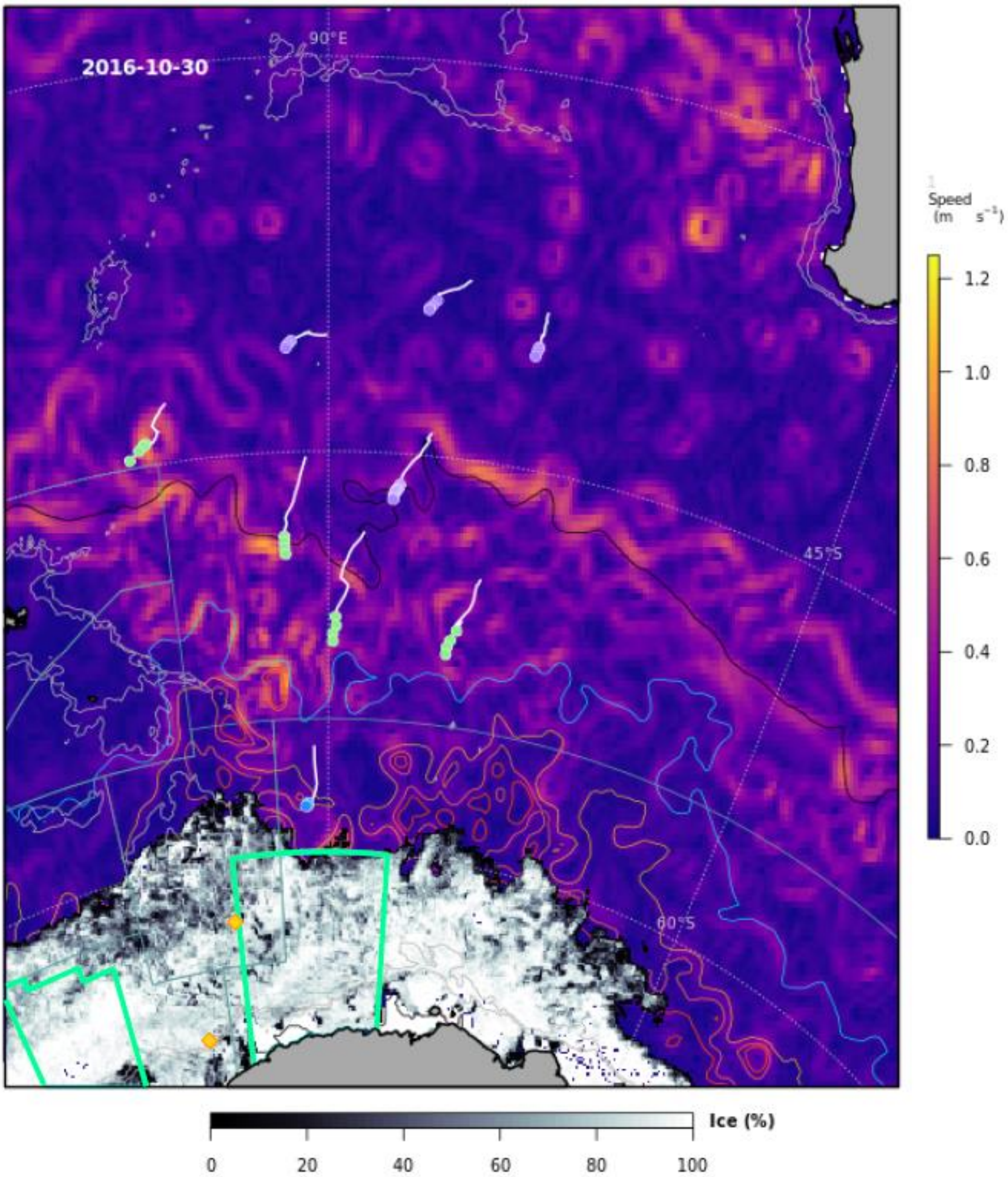

Figure S2.1. Example daily image (30th October 2016) from the Supplementary S2 Tracking Animation (see full explanation for the animation on previous page). Each daily image shows up to four 6-hourly locations per date per individual humpback whale (points), with movements over the 3 days prior shown as a tail (lines). Individual whales are coloured according to sex following Fig. 2 in main manuscript (Female – green, Male – purple, Unknown – blue). Background displays daily sea ice concentration (%) and the absolute magnitude of geostrophic current velocities (m s^{-1}). Also shown are the locations of two acoustic listening stations (yellow diamonds) located at Prydz Bay (2013) and the southern Kerguelen plateau (2014-2017) as part of the Southern Ocean hydrophone network (Miller and Miller, 2018). The relevant areas of the proposed East Antarctic Representative System of Marine Protected Areas (cyan boxes) are also indicated (see main manuscript text).

References

Bestley, S., van Wijk, E., Rosenberg, M., Eriksen, R., Corney, S., Tattersall, K., Rintoul, S., 2018. Ocean circulation and frontal structure near the southern Kerguelen Plateau: The physical context for the Kerguelen Axis ecosystem study. Deep Sea Research Part II: Topical Studies in Oceanography, https://doi.org/10.1016/j.dsr1012.2018.1007.1013.

Cavalieri, D.J., Parkinson, C.L., Gloersen, P., Zwally, H.J., 1996, updated yearly. Sea Ice Concentrations from Nimbus-7 SMMR and DMSP SSM/I-SSMIS Passive Microwave Data, Version 1. [January - February 1997 - 2015]. NASA National Snow and Ice Data Center Distributed Active Archive Center. Boulder, Colorado USA. http://dx.doi.org/10.5067/8GQ8LZQVL0VL. Home page and metadata: http://nsidc.org/data/docs/daac/nsidc0051_gsfc_seaice.gd.html [accessed 29 August 2018].

Kim, Y.S., Orsi, A.H., 2014. On the Variability of Antarctic Circumpolar Current Fronts Inferred from 1992–2011 Altimetry. Journal of Physical Oceanography 44, 3054-3071.

Maslanik, J., Stroeve, J., 1999, updated daily. Near-Real-Time DMSP SSMIS Daily Polar Gridded Sea Ice Concentrations, Version 1. [January - February 2016]. . Boulder, Colorado USA. NASA National Snow and Ice Data Center Distributed Active Archive Center. doi: http://dx.doi.org/10.5067/U8C09DWVX9LM. [accessed 29 August 2018].

Miller BS, Miller EJ. The seasonal occupancy and diel behaviour of Antarctic sperm whales revealed by acoustic monitoring. Scientific Reports. 2018;8(1):5429.

Sokolov, S., Rintoul, S.R., 2009. Circumpolar structure and distribution of the Antarctic Circumpolar Current fronts: 1. Mean circumpolar paths. Journal of Geophysical Research: Oceans 114, C11018.

Sokolov, S., Rintoul, S.R., 2009. Circumpolar structure and distribution of the Antarctic Circumpolar Current fronts: 2. Variability and relationship to sea surface height. Journal of Geophysical Research: Oceans 114, C11019.

van Wijk, E.M., Rintoul, S.R., Ronai, B.M., Williams, G.D., 2010. Regional circulation around Heard and McDonald Islands and through the Fawn Trough, central Kerguelen Plateau. Deep-Sea Res I 57, 653-669.

Weatherall, P., Marks, K.M., Jakobsson, M., Schmitt, T., Tani, S., Arndt, J.E., Rovere, M., Chayes, D., Ferrini, V., Wigley, R., 2015. A new digital bathymetric model of the world's oceans. Earth Space Sci 2, 331-345.

SUPPLEMENTARY S3 – HIDDEN MARKOV MODEL RESULTS

All Hidden Markov Models (HMMs, see Zucchini et al. 2016) investigated were 2-state movement models, nominally representing 'resident' and 'transit' movement behaviours. Models investigated were: 1) basic 2-state model, 2) 2-state with sex-dependent transition probabilities, 3) 2-state with sex-dependent movement parameters and 4) 2-state with sex-dependent transition probabilities and movement parameters.

Model fitting followed an incremental approach, starting from the simplest model (1) with no covariates, and extracting initial parameter estimates from this simplest (nested) HMM for the more complex models (2 and 3); model 4 extracted initial parameter estimates from model 3. All models were fit using the R package *momentuHMM* v1.4.2 (McClintock and Michelot, 2018). Using AIC to compare the four models showed model 3) was supported in favour of the alternative models with fewer or more complex parameterisations (Table S3.1).

We performed multiple imputation analyses to account for errors in the position estimates output from the location filtering state-space model (ssmTMB). This approach repeatedly fits the HMM to multiple realizations of the position process, using the error estimates obtained for each position from the location filter. The step length and turn angle data streams therefore vary among the multiple realizations of the position process, and the pooled inferences across the HMM analyses reflect location uncertainty. We report model parameters calculated as pooled estimates and 95% confidence intervals across n = 100 model fits. All computing was done using the Nectar Research Cloud, a collaborative Australian research platform supported by the National Collaborative Research Infrastructure Strategy (NCRIS).

The most likely state sequence was determined for each fitted model using the Viterbi algorithm (Zucchini et al. 2016), and state sequences, state probabilities and activity budgets (i.e. the proportion of time steps allocated to each state) were calculated using standard multiple imputation formulae (see documentation in McClintock and Michelot, 2018). Plots showing the estimated state-dependent distributions for the step lengths and the turning angles (Fig. S3.1) together with the movement parameter estimates and their 95% confidence intervals (Table S3.2) are given below.

References

McClintock, B.T. and Michelot, T. 2018. momentuHMM: R package for generalized hidden Markov models of animal movement. Methods in Ecology and Evolution 9(6): 1518-1530.

Zucchini, W., MacDonald, I. L., and Langrock, R. 2016. Hidden Markov models for time series: an introduction using R. Second edition. Chapman and Hall/CRC, Boca Raton, Florida, USA.

Table S3.1. Model ranking results for the Hidden Markov Models (HMMs) fitted to humpback whale tracks (N = 5114 locations) investigating sex-related movement patterns. Abbreviations: SL – step length, TA – turning angles, AIC – Akaike Information Criterion.

**2-state movement models**	**Transition probabilities**	**Movement parameters (SL and TA)**	**AIC**	**AIC weights**
1) Basic			43921.67	0
2) Includes sex effects on the state transition probability parameters	~ sex		43854.96	0
3) Includes sex effects on the movement parameters (SL and TA concentration)		~ sex	**42221.09**	**0.889**
4) Includes sex effects on both the state transition probability and movement parameters	~ sex	~ sex	42225.24	0.111

Table S3.2. Estimates of the model movement parameters and their 95% confidence intervals. In each state (resident, transit), μ and σ are the mean and standard deviation of the gamma distribution for the step lengths, respectively; λ and K are the mean and the concentration of the wrapped Cauchy distribution for the turning angles, respectively. The step lengths are measured in kilometres (per 6h time step), and the angles in radians. Results are presented for the 2-state HMM including sex effects on the movement parameters (model 3 in Table S3.1 above, see also Fig. S3.1).

**Parameter**	**Female**	**Male**	**Unknown**
$\mu_{resident}$	12.91 [11.55, 14.26]	5.32 [5.08, 5.57]	5.08 [4.74, 5.42]
$\mu_{transit}$	30.89 [28.70, 33.08]	18.96 [17.63, 20.30]	23.99 [22.16, 25.82]
$\sigma_{resident}$	9.04 [7.83, 10.25]	3.53 [3.30, 3.77]	3.33 [2.97, 3.69]
$\sigma_{transit}$	15.27 [13.70, 16.84]	13.14 [11.98, 14.31]	14.20 [12.28, 16.12]
$\lambda_{resident}$	-3.14 [-3.17, -3.11]	-3.14 [-3.14, -3.14]	-3.14 [-3.16, -3.11]
$\lambda_{transit}$	0.01 [-0.06, 0.08]	-0.01 [-0.12, 0.14]	-0.02 [-0.11, 0.06]
$K_{resident}$	0.07 [0.00, 0.15]	0.00 [0.00, 0.00]	0.00 [0.00, 0.00]
$K_{transit}$	0.62 [0.56, 0.68]	0.38 [0.31, 0.44]	0.60 [0.53, 0.66]

a)

b)

c)

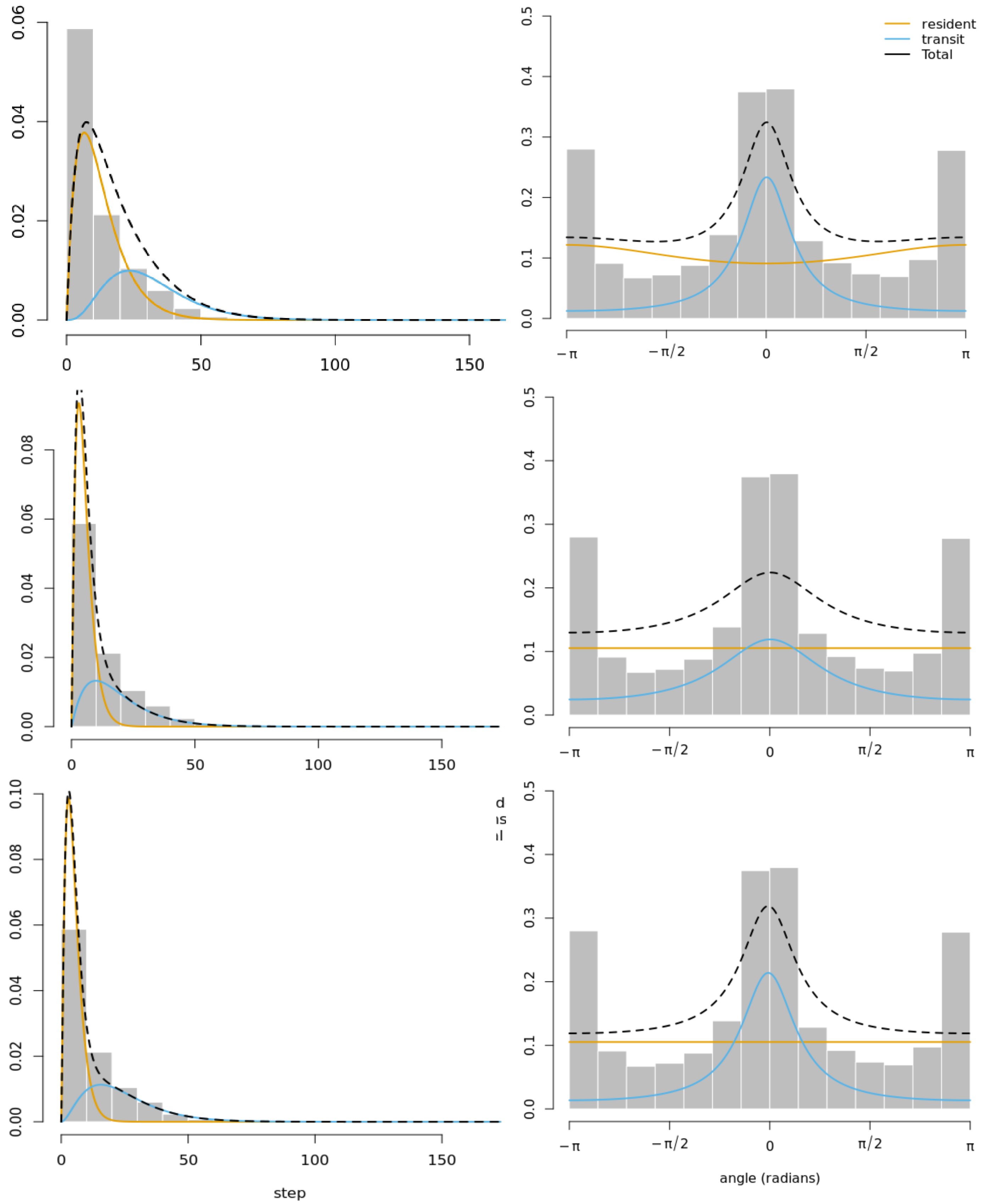

Figure S3.1. Estimated state-dependent distributions for the step lengths (left) and the turning angles (right) for a) female, b) male, and c) unknown sex humpback whales. Histograms show the step lengths and the turning angles of the data, with the estimated state-dependent gamma and wrapped Cauchy densities overlaid. Results are presented for the 2-state HMM that includes sex effects on the movement parameters (SL and TA concentration, model 3 in Table S3.1 above) with model parameter estimates pooled across multiple imputations.

SUPPLEMENTARY S4 – DIVE LOGGER DATA

A small amount of information on vertical movements is available for two migrating Western Australian humpback whales (IDs 131140 and 154875; Fig. S4.1) that were equipped with WC SPLASH10-260C satellite and dive logger tags (see electronic supplementary material S1 for tag deployment details).

The Mk10 instruments were programmed to record pressure every 1 second and compile 6 hourly dive summaries into 14 user-defined depth bins (10; 20; 30; 40; 50; 60; 80; 100; 150; 200; 250; 300; 350; >350m). These tags transmit count information on the total number of dives (Fig. S4.2) and the maximum dive depths (Fig. S4.3) recorded per 6h period, as well as histogram summaries of the maximum dive depths (Fig. S4.4) and the proportion of time-spent-at-depth (Fig. S4.5).

Of particular interest here, the adult female HBW ID131140 had 145 location estimates (99% resident) within CCAMLR statistical area 58.5.1, offshore from the northern Kerguelen plateau. ID131140 crossed 45°S downstream of the northern Kerguelen plateau on October 31 2016 and remained in this area until the tag failed on 6 December (Fig. S4.1).

During this period the 6 hourly summaries show very active vertical movements (Table S4.1). Over 5000 dives were recorded during a reporting period equivalent to approximately 2 weeks (Fig. S4.2). On average, 97 ± 51 dives were recorded per 6h period, with a maximum of 300 reported. Mean maximum dive depths were 83 ± 38 (6 – 180) although deeper dives up to 244 m were reported elsewhere (Fig S4.3).

The summaries by depth bin show this individual exhibited predominantly epipelagic behaviour (Fig S3.4). During this period at the northern KP, HBW ID131140 recorded a median of 30 dives (IQR: 3– 57, maximum: 201) per 6h period in the upper 10m, with a similar number deeper than 50 m (median: 26, IQR: 4 – 49; Table S4.1). On average 50 ± 24% (range: 18 – 90%) of time was spent in the surface 10 m of the water column, and 97.5 ± 8% was within the upper 100 m (Fig. S4.5). HBW ID131140 showed active usage of the upper water column, with maximum time-spent-at depth values above 30% in depth bins 80 to 150 m (maximum value for 80 m: 38%, 100 m: 32%, 150 m: 31%, 200 m: 21%). While the median value for time at depths between 50 to 150m appears relatively low overall at 12% (IQR: 3 – 36%) the upper quartile value indicates that in 25% cases at least 36% of the 6h period was spent at these depths, i.e. roughly equivalent to just over 2h per 24 h reported.

Unfortunately, the dive logger for HBW ID154875 provided much less data overall (total of 4359 dives) and very little information within the Southern Ocean. Mean maximum dive depths for this individual (Table S4.1, Fig. S4.3) were somewhat deeper overall at 109 ± 65 (7 – 350; n = 182 reporting periods), and similar south of 45°S at 98 ± 73 (7 – 264; n = 34). Otherwise, only six 6-hourly summaries are available south of 45°S (Figs. S4.2, S4.4 and S4.5).

Table S4.1. Summary of dive information available for migrating HBWs. Second column provides data summaries for adult female HBW ID131140 specifically whilst in the vicinity of the northern Kerguelen plateau (KP), Southern Ocean. All data are compiled per 6h period onboard the dive loggers, excepting totals given across all reporting periods. Values reported as mean ± SD (min – max) followed by the median (interquartile range) values, i.e., 50% (25 – 75%). Abbreviations: TAD – time-at-depth.

	**ID 131140 Full time-series 4 Oct – 6 Dec 2016**	**ID 131140 Northern KP 31 Oct – 6 Dec 2016**	**ID 154875 Full time-series 15 Sep – 7 Nov 2016**
**No. of dives**			
Total recorded	8366	5229	4359
No. 6h reporting periods	98 (approx. 24.5 d)	54 (approx. 13.5 d)	59 (approx. 15d)
Mean no. dives per 6h	85 ± 44 (27 – 300)	97 ± 51 (38 – 300)	74 ± 32 (19 – 161)
Median no. dives per 6h	76 (52 – 105)	83 (64 – 121)	69 (51 – 93)
**Maximum dive depths (m)**			
No. 6h reporting periods	231 (approx. 58 d)	123 (approx. 31 d)	182 (approx. 45.5 d)
Mean max. dive depth	92 ± 45 (6 – 244)	83 ± 38 (6 – 180)	109 ± 65 (7 – 350)
Median max. dive depth	90 (66 – 125)	80 (63 – 108)	104 (62 – 147)
**Binned maximum depths (m)**			
Mean no. dives upper 10m	32 ± 35 (0 – 201)	40 ± 45 (0 – 201)	21 ± 21 (0 – 81)
Median no. dives upper 10m	23 (9 – 40)	30 (3 – 57)	13 (7 – 29)
Total no. dives upper 10m	3156	2166	1258
Mean no. dives >50 m	24 ± 23 (0 – 71)	28 ± 24 (0 – 71)	18 ± 14 (0 – 51)
Median no. dives >50 m	17 (4 – 42)	26 (4 – 49)	12 (7 – 26)
Total no. dives >50 m	2353	1495	1083
**Binned time-at-depth (%)**			
Mean %TAD upper 10m	48 ± 24 (8 – 96)	50 ± 24 (18 – 90)	30 ± 17 (11 – 87)
Median %TAD upper 10m	43 (25 – 71)	56 (25 – 71)	26 (19 – 35)
Mean %TAD 50–150 m	19 ± 20 (0 – 68)	20 ± 21 (0 – 68)	22 ± 18 (0 – 76)
Median %TAD 50–150 m	10 (3 – 34)	12 (3 – 36)	16 (9 – 28)

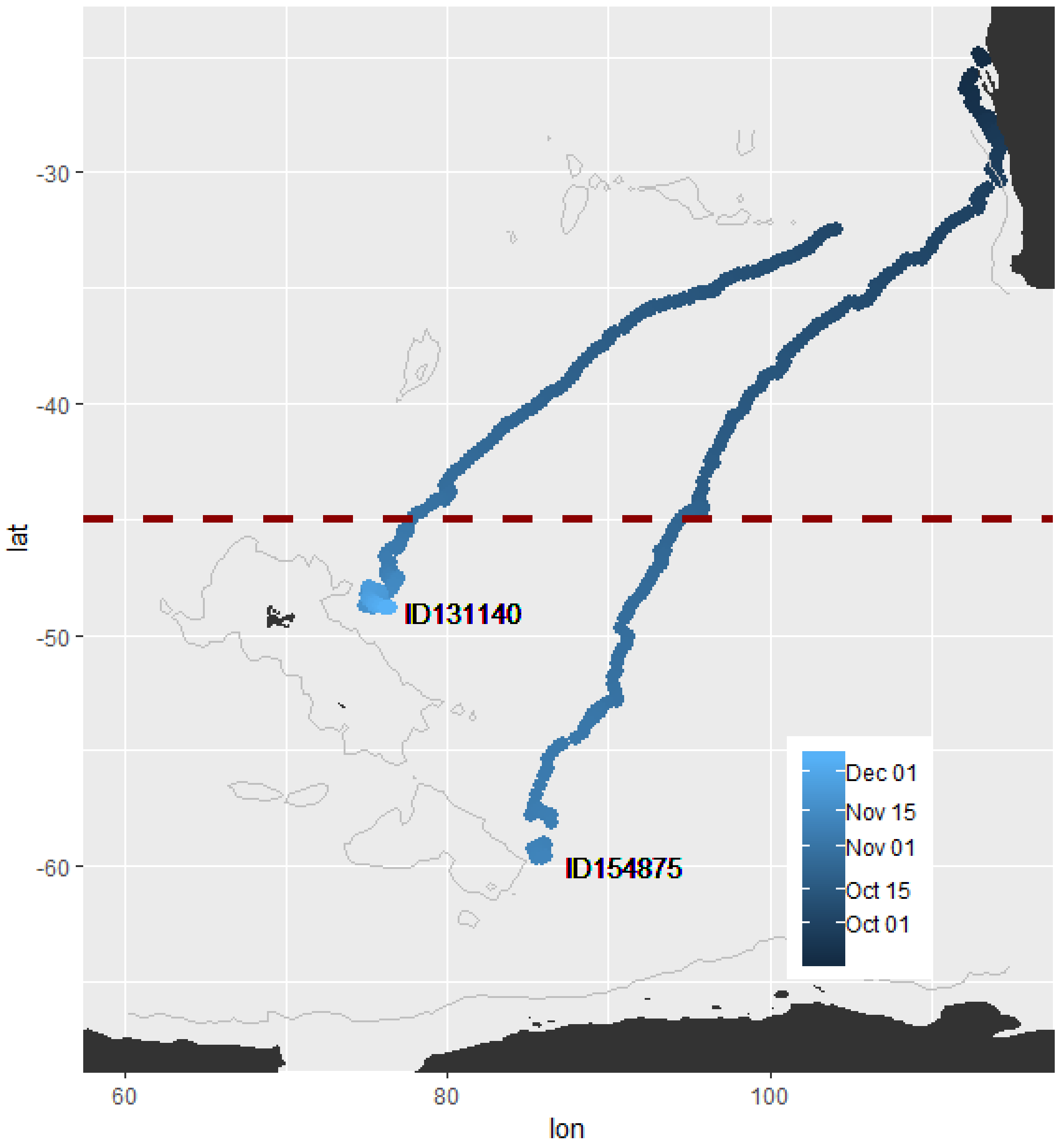

Figure S4.1. Map showing the Southern Ocean migration pathways for two WA humpback whales equipped with dive logger tags. Locations are coloured by date. Red dashed line marks 45°S. Bathymetric contour given at 2000 m.

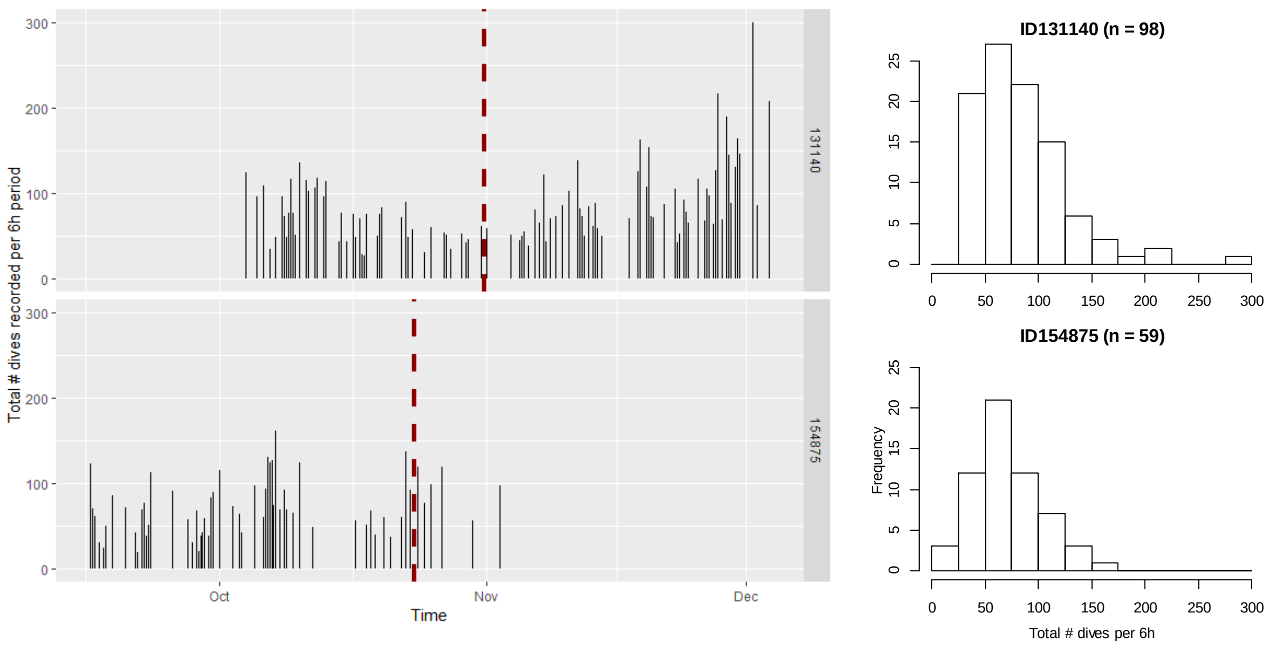

Figure S4.2. Count information on the number of dives performed by HBWs per 6h period. Data shown as time-series with each value representing a 6h period (left-hand panels), and compiled into an overall histogram (right-hand panels; n indicates the number of reporting periods). The total number of dives reported by ID131140 was N = 8366 and by ID154875 was N = 4359, respectively (Table S4.1).

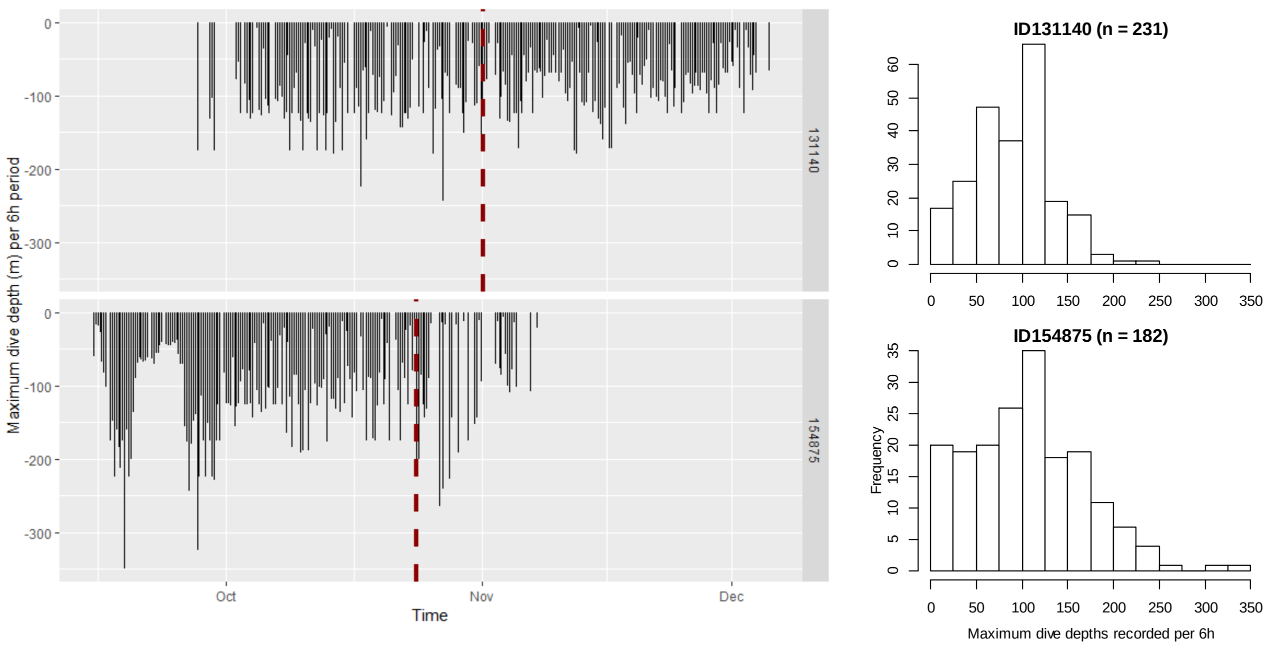

Figure S4.3. Summary information on the maximum dive depths (m) recorded by WA HBWs per 6h period. Data shown as time-series with each value representing a 6h period (left-hand panels), and compiled into an overall histogram (right-hand panels, noting different y-axes). n indicates the number of 6h reporting periods.

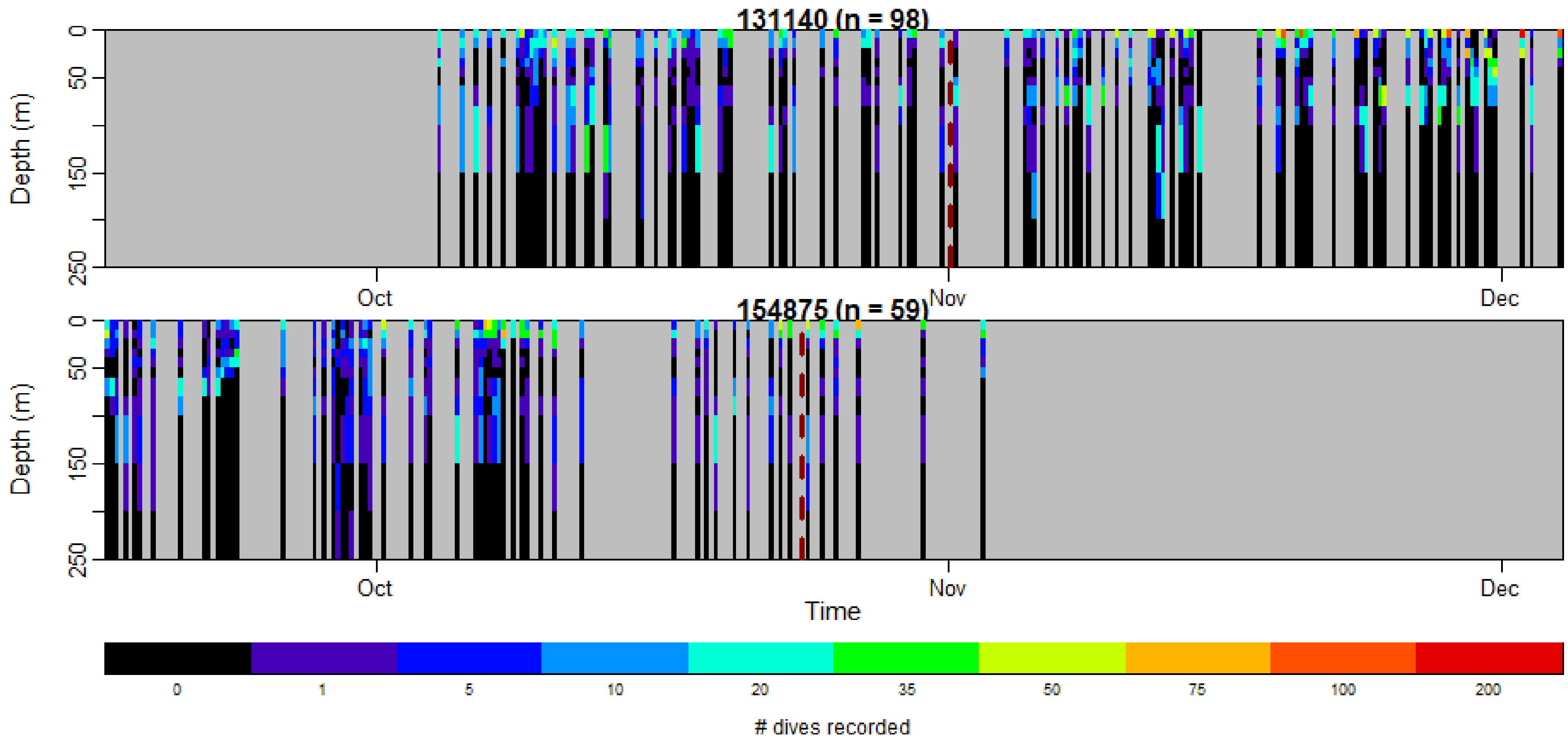

Figure S4.4. Time-series showing HBW maximum dive depths (m) summarised as histograms per 6h period (n = number of 6h periods reported). Dive data are summarised onboard the dive loggers in user-defined depth bins (see text, upper 250 m shown here). Red dashed line marks the date at which the migration path of each individual crossed south of 45°S (see Fig. S4.1). Grey shading indicates no data for these periods.

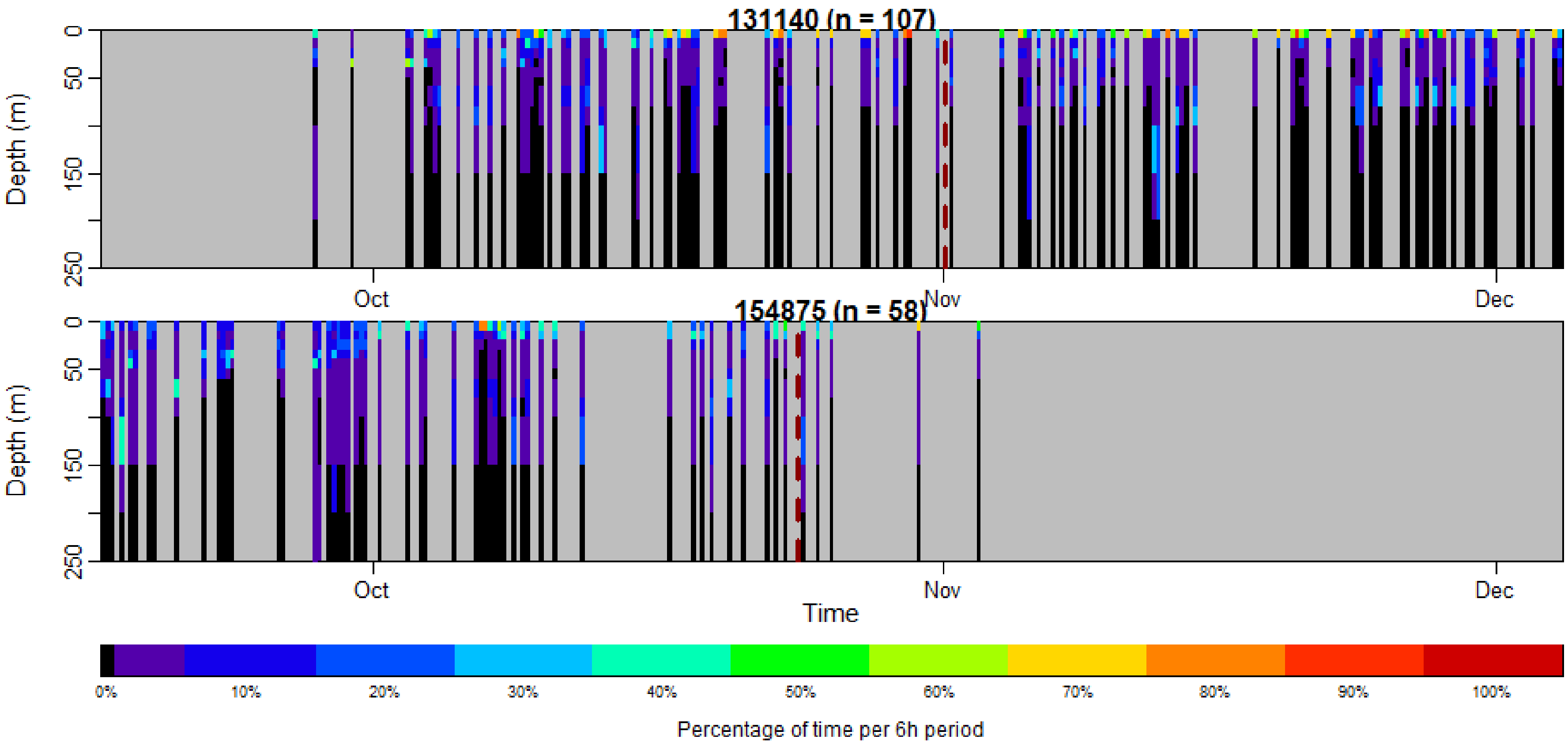

Figure S4.5. Time-series showing the percentage of time-spent-at-depth per 6h period for migrating HBWs (upper: ID131140, and lower: ID154875). Results presented as in Fig. S4.4 above.
